# A self-healing electrocatalytic system via electrohydrodynamics induced evolution in liquid metal

**DOI:** 10.1038/s41467-022-35416-w

**Published:** 2022-12-09

**Authors:** Yifeng Hou, Fengyan Wang, Chichu Qin, Shining Wu, Mengyang Cao, Pengkun Yang, Lu Huang, Yingpeng Wu

**Affiliations:** State Key Laboratory of Chem/Bio-Sensing and Chemometrics, Advanced Catalytic Engineering Research Center of the Ministry of Education, College of Chemistry and Chemical Engineering, Hunan University, Changsha, 410082 P. R. China

**Keywords:** Electrochemistry, Electrocatalysis, Materials for energy and catalysis, Electrocatalysis, Electrocatalysis

## Abstract

Catalytic deterioration during electrocatalytic processes is inevitable for conventional composite electrodes, which are prepared by depositing catalysts onto a rigid current collector. In contrast, metals that are liquid at near room temperature, liquid metals (LMs), are potential electrodes that are uniquely flexible and maneuverable, and whose fluidity may allow them to be more adaptive than rigid substrates. Here we demonstrate a self-healing electrocatalytic system for CO_2_ electroreduction using bismuth-containing Ga-based LM electrodes. Bi_2_O_3_ dispersed in the LM matrix experiences a series of electrohydrodynamic-induced structural changes when exposed to a tunable potential and finally transforms into catalytic bismuth, whose morphology can be controlled by the applied potential. The electrohydrodynamically-induced evolved electrode shows considerable electrocatalytic activity for CO_2_ reduction to formate. After deterioration of the electrocatalytic performance, the catalyst can be healed via simple mechanical stirring followed by in situ regeneration by applying a reducing potential. With this procedure, the electrode’s original structure and catalytic activity are both recovered.

## Abstract (web summary)

Self-healing electrodes may offer opportunities to prevent catalyst deterioration during electrocatalytic reactions. Here the authors demonstrate a self-healing system by exploiting electrohydrodynamics induced evolution of Bi in liquid metal and apply this to CO_2_ electroreduction to formate.

## Introduction

**Fig. 1:**
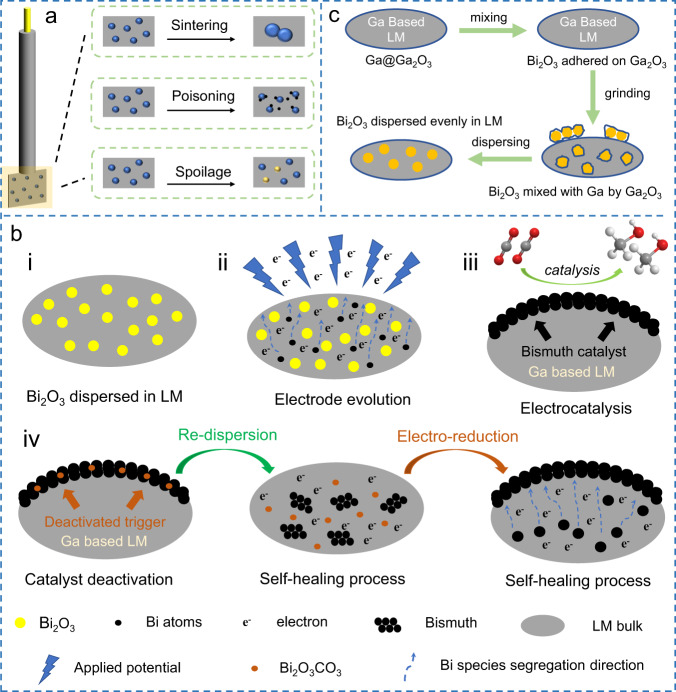
Schematic diagram of traditional electrode deterioration mechanism and the preparation and healing mechanism of the liquid metal electrode. **a** Schematic diagram of the deterioration mechanism for conventional electrodes, including sintering, poisoning, and spoilage in sequence. **b** Schematic diagram of LM electrode in different states or processes. i Bi_2_O_3_ dispersed LM electrode. ii The evolved state induced by electrohydrodynamics. iii The electrocatalytic CO_2_RR on the evolved electrode. iv The self-healing process of the deactivated Bi catalyst in LM sea, including re-dispersion and electrochemical reduction. **c** Schematic diagram for the preparation of Bi_2_O_3_–LM mixture.

Electrocatalysis can efficiently convert raw materials into chemicals by renewable electricity, which attracts great attention in recent years^1,2^. However, electrode/catalyst deterioration is a common problem that reduces catalytic efficiency and further results in additional costs^3,4^. Traditional electrocatalysts are loaded on rigid current collectors (e.g., glassy carbon, carbon paper, and foamed metal) and decay in their catalytic performance by sintering, poisoning, spoilage, or losing active sites during the long-term process (Fig. 1a)^3^. The existing solutions for such deterioration are limited to improving the intrinsic stability or replacing the catalyst. However, these methods are costly and complicated^5^.

Room temperature liquid-metal (LM) Ga and its eutectic alloy (e.g., Ga-In, Ga-Sn, or Ga-In-Sn) have aroused the enthusiasm of researchers in recent years^6,7^. Due to its conductivity, fluidity, flexibility, and biocompatibility progresses based on LM have been developed in different areas, such as battery^8,9^, flexible intelligent device^10^, self-healing materials^11^, and biological drug delivery^12^. The fascinating properties of LM also make it a candidate for catalysis^13–20^, or to be used to fabricate nano catalysts^21,22^. The fluidity characteristics endow LM with the fluid feature of water, so LM can act as a solvent to disperse catalytic particles. The free electrons in LM can form an electron sea which provides abundant sites to promote reactions inside or on LM surface^17,23^. What is more, the electrohydrodynamic effect can lead to the deformation or turbulence of LM under an electric field^24–26^. By this effect, integrated catalysts in LM can be easily switched between the matrix and surface of LM, providing the feasibility for the construction/healing of the catalyst.

Herein, a concept of a self-healing electrocatalytic system was proposed based on an LM electrode. As proof of concept, Bi_2_O_3_ particles were dispersed into the LM matrix as a precursor of the catalyst (state i, Fig. 1b), then a negative potential was applied to actuate the evolution of the precursor. Electrons in LM reduced Bi_2_O_3_ to a highly dispersed Bi atoms intermediate state (state ii, Fig. 1b). Then, electrohydrodynamics triggered the segregation of Bi atoms from the LM substrate to its surface, where catalytic bismuth nanosheets were generated. The evolved electrode was used to perform the electrochemical CO_2_ reduction reaction (CO_2_RR) (state iii, Fig. 1b). In durability tests, catalytic bismuth suffered a deterioration after a long service time. However, by a simple re-dispersion and reconfiguration by applying a negative potential, the deactivated bismuth can be self-healed in the LM sea and segregated on the LM surface again (process iv, Fig. 1b). After this process, both structure and electrochemical performance of the electrode can be healed.

## Results

### Electrohydrodynamic evolution in LM sea

Benefited by the liquid fluidity, other materials can be dispersed into LM medium^23,27^. Here, Bi_2_O_3_ as a catalytic precursor was uniformly dispersed in the LM matrix by simple grinding (Fig. 1c and Supplementary Fig. 1). Note this process was only a physical dispersion, and no alloying or chemical reaction happened. And the LM system was kept in a liquid state during the entire experiment. The behavior of fluids in liquid dielectrics under electric fields can be studied by electrohydrodynamics^28^. In our work, the Bi_2_O_3_–LM mixture was loaded in a 3D printed catalytic cell (Supplementary Fig. 2), and electrohydrodynamic evolution was performed by applying a negative potential in the KHCO_3_ electrolyte.

As a common electrochemical diagnosis method, cyclic voltammetry (CV) was adopted to preliminarily study the possible reactions in the Bi_2_O_3_–LM system under an electric field (detailed in the Methods part: CV diagnosis). Within a tentative potential range from −1 V to 0 V (unless specified, the potentials are all compared vs. the reversible hydrogen electrode (RHE)), the current density decreased as the CV cycles increase and finally stabilized after 400 cycles. (Supplementary Fig. 3a, b). During this process, some insoluble black products gradually appeared on the LM electrode surface (Supplementary Fig. 2c). From the change of CV curves, it can be speculated that there is a progressive and gradually stabilized reaction. To find out the electrochemical reaction during this process, a control experiment was carried out with pure Ga under the same conditions (Supplementary Fig. 4). By contrasting the different CV curves (Supplementary Figs. 3 and 4) and the phenomena occurred on the LM surface, the electrochemical event can be speculated that Bi_2_O_3_ was electrochemically reduced to bismuth and emerged on LM surface (detailed in the discussion after Supplementary Fig. 4).

**Fig. 2:**
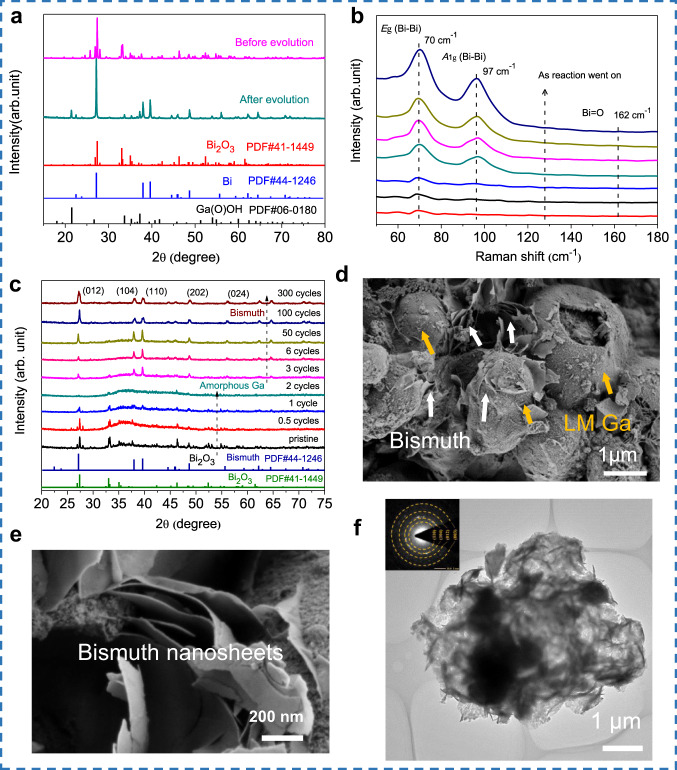
Composition and morphology characterizations of bismuth products before and after electrochemical evolution. **a** XRD patterns for raw material Bi_2_O_3_ and the collected black products on the LM surface after CV diagnosis. **b** In situ Raman spectra for detecting the surface transformation of LM electrode as negative potential applied. **c** In situ XRD patterns for revealing the pathway and phase transformation of Bi species by electrohydrodynamic evolution. **d** SEM image of collected Bi from LM surface. **e** Partially enlarged image of (**d**). **f** TEM image of generated bismuth by electrohydrodynamic evolution, insert graph is fast Fourier transform (FFT) pattern of the crystalline bismuth. Source data are provided as a Source Data file.

To get a further understanding of this process, X-ray diffraction (XRD) was applied to verify the reactions in this Bi_2_O_3_-LM system. The Bi_2_O_3_ used here was confirmed as α-Bi_2_O_3_ (purple line, Fig. 2a). After evolution, the black products collected from the LM surface were confirmed as crystallized bismuth (green line, Fig. 2a). None of the Bi_2_O_3_ characteristics peaks were detected after evolution^29^. These results confirmed that the arisen Bi originated from Bi_2_O_3_ in the LM matrix. In-situ microscopy captured the microscopic process of bismuth segregation on the LM surface from the LM matrix (Supplementary Movie 1 and Supplementary Fig. 5). What is more, no Ga–Bi alloy peaks were detected, confirming no alloy reaction between Ga and Bi, as Ga–Bi alloying usually needs high temperature^30^. Slight Ga(O)OH (green line, Fig. 2a) can also be detected, as the broad CV range (−1 V to 0 V) may lead to the oxidation of Ga, to avoid the affection of Ga(O)OH, we choose −1 V to −0.6 V in the following test.

The morphology of generated bismuth was confirmed by SEM. Two-dimensional bismuth nanosheets (2D Bi NSs) were observed with layered structures grown on the Ga surface (Fig. 2d, e). The EDS mapping proved the nanostructure consists of Bi covered on Ga (Supplementary Fig. 6). The structure of bismuth was also confirmed by TEM (Fig. 2f), with an illustration of a fast Fourier transform (FFT) pattern on the left top, showing the structure of crystal bismuth.

Although there are several works on galvanic replacement of metal/metal acid group ions on Ga surface^22,26,31,32^, the electrochemical evolution of metal oxide in LM sea was achieved differently in our work. We consider this evolution from metal oxide (Bi_2_O_3_) to pure metal (Bi) as not a simple galvanic replacement that occurred between the electrolyte and LM interface. The evolution includes the electrochemical reduction from Bi_2_O_3_ to Bi atoms and the following crystallization of Bi by electrohydrodynamic-derived segregation. To prove this, in-situ Raman spectrum and in-situ XRD were introduced.

During the testing process of in-situ Raman spectroscopy, two peaks gradually emerged at 70 and 91 cm^−1^, which represented the *E*_g_ and *A*_1g_ stretching modes of Bi–Bi bonds, respectively (Fig. 2b)^29,33^. The increased intensity of these Raman peaks indicated the accumulation of bismuth on the LM surface as the reaction went on, which is consistent with the XRD results above (Fig. 2a). Because Raman spectroscopy is a surface-focused scanning, the lack of the Bi=O signal suggested there is no Bi_2_O_3_ appeared on LM surface during the entire testing process. That is to say, the detected Bi on the LM surface comes from the LM matrix. So, the possibility can be excluded that Bi_2_O_3_ first migrates from the LM matrix to the LM surface and then is reduced. Actually, the entire evolution of Bi_2_O_3_ occurred inside LM (Supplementary Fig. 7).

In-situ XRD revealed a transient intermediate state of Bi products in LM sea during the electrohydrodynamic evolution. As shown in Fig. 2c, the electrohydrodynamic evolution of Bi species in LM sea can be divided into three stages: (1) Before CV scanning, the characteristic diffraction of dispersed Bi_2_O_3_ and amorphous features of Ga (the bulge peak at ~36°)^34^ can be detected (pristine line, Fig. 2c). (2) As the CV scanning went on, peaks of Bi_2_O_3_ perished quickly, leaving only amorphous characteristics of Ga (the curves from 0.5 to 2 cycles, Fig. 2c). And (3) the characteristic diffraction peaks of crystalline bismuth gradually emerged with an increased (012) plane (the curves from 3~300 cycles, Fig. 2c). Note that no segregation phenomenon of bismuth (i.e., black products on the surface of LM) can be observed until the 3rd stage. In other words, there should be an undetectable transition state of Bi species before the construction of crystal bismuth. To confirm this, we dissected the LM electrode after two cycles of CV scanning, to check the interior composition of the electrode. However, by XRD result, no Bi_2_O_3_-related peaks can be detected from the interior electrode (Supplementary Fig. 8). This indicates an intermediate reduction state of Bi element in LM sea during the evolution process: under the electric field, Bi_2_O_3_ was reduced into highly dispersed Bi atoms which can not be detected by XRD before the third cycle (stage 1 and 2), then the dispersed Bi atoms migrated from the matrix of LM and segregated on LM surface to form crystal bismuth (stage 3).

Considering that the reduction potential of Bi is more positive than that of Ga, the galvanic replacement may also occur in the LM sea at the same time. Additional experiments were carried out (detailed in Supplementary Fig. 9 and followed discussions), and the results suggest that galvanic replacement only happened at the LM–solution interface and can not happen in the LM matrix. The rate of this surface galvanic replacement is very slow, with only 1/68 of the electrohydrodynamic-induced evolution. Overall, the applied potential provides the main driving force for Bi_2_O_3_ reduction and segregation in LM.

### The electrohydrodynamic induced non-equilibrium driving force for bismuth segregation and morphography controlling

In fact, the excessive Bi atoms generated by the electrochemical reduction in the LM matrix are thermodynamically unstable, tending to segregate spontaneously^35^. However, the spontaneous driving force is not strong enough to cause rapid segregation, which is proved by the following experiments: as the applied electric field can be controlled, the reduction process of Bi_2_O_3_ was precisely adjusted to a highly dispersed atoms state by a transient electric potential (before stage 3 in in situ XRD part discussed above) but without the following segregation, then stopped the voltage applying. In this way, we can clarify whether electricity has any effects on driving bismuth segregation and controlling its morphology. In other words, the question can be cleared up whether the segregation of bismuth can happen without the electrohydrodynamic driving force. As shown in Supplementary Fig. 10, without the voltage applying, subsequent spontaneous segregation was very difficult. After 24 and 48 h, only slight black spots appeared on the LM surface, far less than the amount caused by continuous electrohydrodynamic driving in 2 h.

We can consider this electrohydrodynamic process from the segregation formula^36^

1 $${(\frac{{n}_{A}}{{n}_{B}})}_{{{{{{\rm{surface}}}}}}}={(\frac{{N}_{A}}{{N}_{B}})}_{{{{{{\rm{matrix}}}}}}}{e}^{\frac{\varDelta {{{E}}}_{A}-\varDelta {{{E}}}_{B}}{{k}_{B}T}}$$

where *ΔE*_A_ and *ΔE*_B_ represent segregation energy of solute (Bi) and solvent (Ga); *n*_A_, *N*_A_ and *n*_B_, *N*_B_ represent the surface and matrix atoms number of solute and solvent; *k*_*B*_ represents the Boltzmann constant; *T* represents absolute temperature.

When Δ*E*_A_ > Δ*E*_B_ (solute segregation energy is larger than solvent), the *n*_A_/*n*_B_ > *N*_A_/*N*_B_, solute atoms spontaneously segregated in the solvent matrix. In our experiment, with no potential applied, the Bi atom’s spontaneous segregation was very slow, indicating that a driving force is required to increase the segregation energy for Bi atoms during the segregation and crystallization process. In fact, when electric field is applied on an LM electrode in non-Faraday scope, the relationship between voltage and interface energy is described as an electrocapillary equation^37,38^:

2 $$\gamma (V)={\gamma }_{0}-\frac{1}{2}C{(V-{V}_{0})}^{2}$$

where *γ*_0_ is the interfacial energy with initial status in open circuit voltage (OCP); C is the capacitance of the double layer; *V* and *V*_0_ are the applied electric potential and initial potential at *γ*_0_, respectively; *γ*(*V*) is the interfacial energy when potential *V* was applied.

By adjusting the applied potential, the interfacial energy *γ*(*V*) between electrolyte and LM can be controlled by applied *V* (Eq. 2). On the other hand, if considering these electrohydrodynamic driven events from the crystal nucleation and growth, according to the Gibbs–Thomson equation^39,40^:

3 $$\varDelta G=4\pi {r}^{2}\gamma -\frac{4}{3}\pi {r}^{3}\frac{{{{{{\rm{RT}}}}}}}{{{{{{\rm{V}}}}}}}\,{{{{{\mathrm{ln}}}}}}\,c/{c}_{\infty }$$

where *ΔG* represents the Gibbs free energy of the new phase; *c*/*c*_∞_ represents the degree of supersaturation; *γ* represents the interface energy of two-phase, *V* and *r* represent the newly generated atomic volume and radius, respectively.

When the system reaches a critical state of crystallization, *r* = r*, *∂∆G/∂r* = 0, thus:

4 $${r}^{* }=\frac{2\gamma V}{{{{{{\rm{RTln}}}}}}c/{c}_{\infty }}$$

5 $${{\mbox{∆G}}}^{{{\mbox{ * }}}}{{\mbox{=}}}\frac{{{\mbox{16}}}{\pi}{\gamma}^{{{\mbox{3}}}}{{\mbox{V}}}^{{{\mbox{2}}}}}{{{\mbox{3}}}{{{\mbox{(}}}{{\mbox{RT}}}{{\mbox{ln}}}{{\mbox{c}}}{{\mbox{/}}}{{\mbox{c}}}_{{{\mbox{∞}}}}{{\mbox{)}}}}^{{{\mbox{2}}}}}$$

r^*^ represents the new phase nucleation radius, ΔG^*^ represents the Gibbs free energy of critical nucleation, which is also the nucleation activation energy for new phase formation.

According to Eq. (5), interfacial energy *γ* is a positive correlation to the nucleation activation energy ΔG^*^; when a more negative potential was applied, the interfacial energy between the LM and aqueous solution became larger (Eq. 2), as the LM droplet electrode tends to have a higher curvature radius. In this situation, however, the interfacial energy between LM with the newly formed bismuth was smaller, which leads to a smaller critical nucleation energy of the new bismuth phase. This reasonably explained why the segregation of bismuth from the LM matrix got easier after applying an electric field in this system.

As the applied electric field can directly modulate the nucleation energy of crystalline bismuth, different modes of potential were applied to control the segregated bismuth morphology. To study the influence of different segregation behavior, three voltage modes (including constant voltage polarization (CVP), pulsing oscillation polarization (POP), and triangle potential scanning (TPS)) were applied to the LM electrodes. The results proved the non-equilibrium state caused by different Gibbs free energy changes can lead to different morphologies of the generated bismuth^41^.

**Fig. 3:**
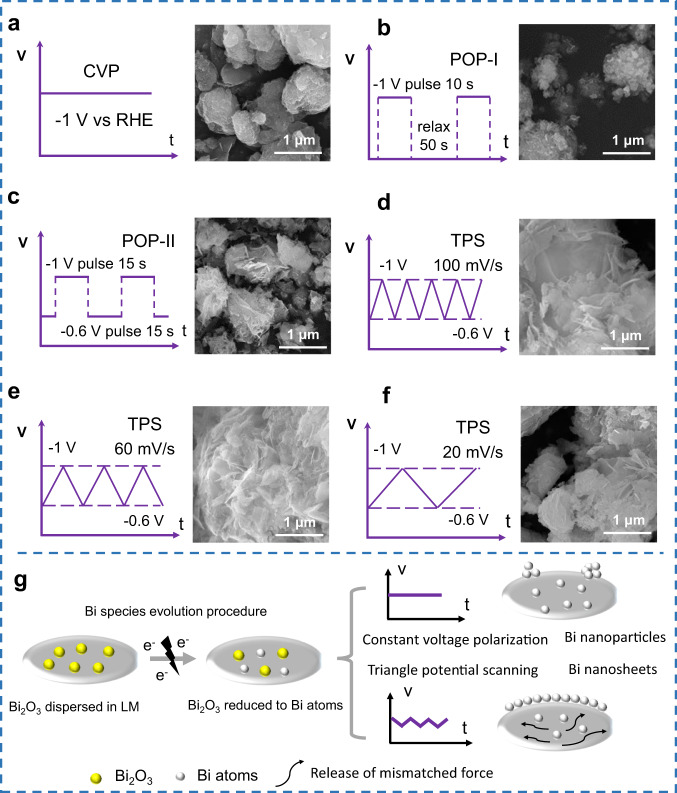
Morphology controlling of generated Bi by different electrochemical modes with corresponding images and mechanism discussion. **a** Constant potential polarization (constant −1 V potential applied, CVP). **b** Pulsing oscillation polarization I (pulsing 10 s at −1 V then relaxing 50 s, total time keeps 1 min for a cycle, POP-I). **c** Pulsing oscillation polarization II (the potential was forced to oscillate between −1 V for 15 s and −0.6 V for 15 s, POP-II). **d**–**f** Triangle potential scanning (from −1 V to −0.5 V, with different scan rates **d** 100 mV s^−1^, **e** 60 mV s^−1^, and **f** 20 mV s^−1^, respectively. TPS). **g** Schematic diagram of a mechanism for morphological differences by different electrohydrodynamic derived evolution.

When a CVP (detailed in Method parts: different potential modes for controlling the segregated bismuth morphology) mode was applied, the segregated bismuth was prone to form nanoparticles (Fig. 3a). In POP modes, two different pre-set configurations were tested and showed different results: for first configuration (Fig. 3b), intermittent pulsing stays at −1 V for 10 s by a shelve time of 50 s for charge relaxation (POP-I). In this situation, most products are granular particles with very few nanosheets. In another configuration, a forced oscillation change was applied between −1 V to −0.6 V by pulsing oscillation polarization, and without any relaxation (POP-II), some layered structures began to emerge in this configuration (Fig. 3c). Actually, when Bi atoms are segregated and crystallized from the matrix of LM, CVP mode generated constant surface energy, which led to a stable Bi nucleation energy, further resulted in Bi forming nanoparticles under such a stable condition because of the uniform driving force. While in POP-I mode (divided into pulsing stage and pausing stage), the situation of Bi within the first 10 s during −1 V applying is the same as CVP. In the pausing stage (relaxed 50 s), although the electric field was removed, the electrode potential of the LM changed a little (Supplementary Fig. 11), which resulted in no significant change in LM surface energy because it was controlled by the applied potential (Eq. 2). When the potential continued, Bi grew again and repeated the former process, so the Bi morphologies produced by POP-I and CVP are quite similar. In POP-II, however, the potential was forced to oscillate between −1 V and −0.6 V, which resulted in a sudden change in the surface energy of LM. This non-equilibrium perturbation led to uneven stress during the growth of Bi, hence irregular sheet-like structures were observed.

At last, TPS mode was employed within a potential window from −1 V to −0.6 V at different scan rates of 100–20 mV s^−1^ (Fig. 3d–f). Compared with CVP and POP, TPS can provide a continuous stable non-equilibrium change, rather than a sudden changing as POP-II or no change as CVP. Not unexpectedly, the 2D Bi NSs dominated the main morphology in this mode. At a low scan rate (20 mV s^−1^), the Bi NSs possessed larger and thicker size, while at large scan rates (100 mV s^−1^), non-equilibrium drove rapid segregation kinetics, which made 2D Bi NSs unfavorable to grow larger, so the morphology was inclined to be smaller and thinner.

The different driving force from electrohydrodynamics will lead to different morphology of bismuth, which is very similar to graphene growth by epitaxy (Fig. 3g and Supplementary Fig. 12). The difference is that the driving force for graphene epitaxy is temperature, while in the LM system, electrohydrodynamics induced segregation /crystallization dominated the process. In epitaxy, if the lattice stress between substrate and growth atoms is hard to release, 3D growth of graphene islands will occur (Wolmer-weber mode). Otherwise, 2D graphene is inclined to form (Frank van der Merwe mode)^42,43^. The difference in epitaxy can be a good guide to understanding our experimental results: on CVP and POP modes, although Ga is amorphous, the mismatching force from Bi atoms substrate can not release timely (as these two modes can not continuously modulate the surface energy between LM with newly generated bismuth), which resulted in atomic uneven nucleation to form Bi nanoparticles. In a TPS way, the surface energy of LM substrate with bismuth was adjusted by time and continuously changed potential, causing this mismatched force to be released in the other direction^43^, so the main morphology was Bi nanosheets. Supplementary Movies 2–4 clearly depicted the actuation behavior and the LM surface state changing in three different modes, which demonstrated the regulation of LM surface energy by the three different potential modes in detail.

### Electrocatalysis CO_2_RR and self-healing electrode

**Fig. 4:**
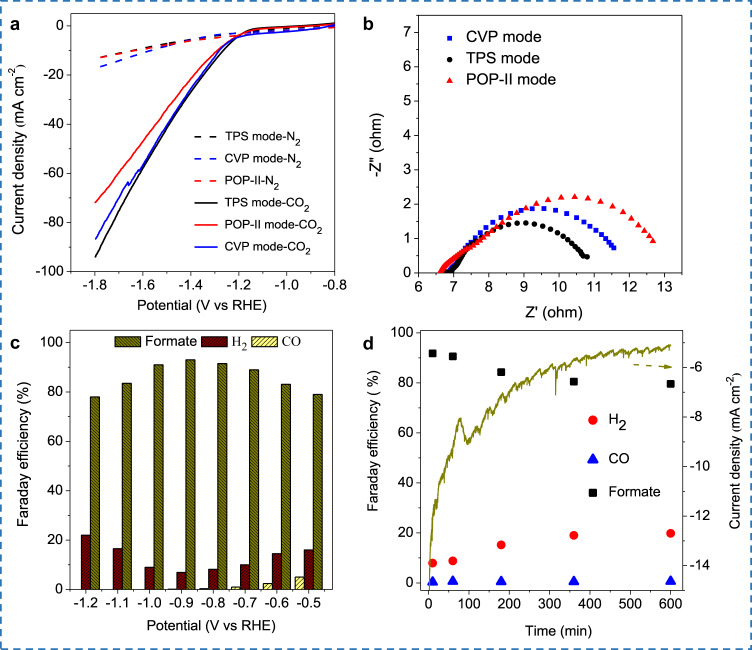
Electrochemical data for evolved Bi-based LM electrode. **a** LSV curves for Bi-based LM electrode in N_2_ or CO_2_ saturated 0.5 M KHCO_3_ derived by CVP, POP, and TPS modes. **b** Electrochemical impedance comparison of Bi-based LM electrodes derived by CVP, POP, and TPS modes. **c** Faraday efficiency (FE) on TPS derived LM electrode from −1.2 V to −0.5 V. **d** The current density and FE of CO_2_RR during 10 h of electrolysis with the TPS evolved LM electrode. Source data are provided as a Source Data file.

The evolved Bi-based LM electrode was used for electrocatalytic CO_2_RR, as Bi or its alloy exhibits excellent CO_2_ electrocatalytic activity and selectivity^29^. For example, Bi-Sn nanoparticles were produced by ultrasound liquid alloy and employed as a catalyst for CO_2_RR^21^. Figure 4a depicted the LSV curves for CO_2_RR by these Bi-based LM electrodes derived from three different voltage modes discussed above (i.e., CVP, POP-II, and TPS).

All the cases exhibited more negative currents in CO_2_-saturated electrolytes than those in N_2_, confirming the excellent CO_2_RR catalytic ability of the Bi-based LM electrode^16,21^. In fact, Bi had a poor ability of HER, when it was loaded on the LM surface, HER was suppressed while good CO_2_RR performance was obtained^44,45^. The electrode with the best catalytic performance was obtained by the TPS mode. This should be attributed to the TPS-produced 2D Bi NSs can provide more catalytic sites, enhance the adsorption ability of intermediates, and further enhance the intrinsic activity^46,47^. Moreover, as the TPS provided a stable Bi NSs loading, which is more suitable for CO_2_RR. POP may lead to a disordered growth of bismuth, which further led to harsh contact between bismuth with substrate Ga. Compared with the CVP and TPS, the electrode experienced POP mode exhibited the largest impedance (Fig. 4b). TPS derived electrode with the best CO_2_RR catalytic performance was used to test the FE from -0.5 V to −1.2 V, which was quantitatively analyzed with nuclear magnetic resonance (NMR) and gas chromatography (GC) (Supplementary Figs. 13 and 14). The products were determined to be formate, CO, and H_2_. The FE of formate is almost >80% within the whole potential window, and CO was only detected at the potential more negative than −0.7 V. The whole process presented a volcanic trend, and the maximum FE is ~93% at −0.9 V (Fig. 4c).

**Fig. 5:**
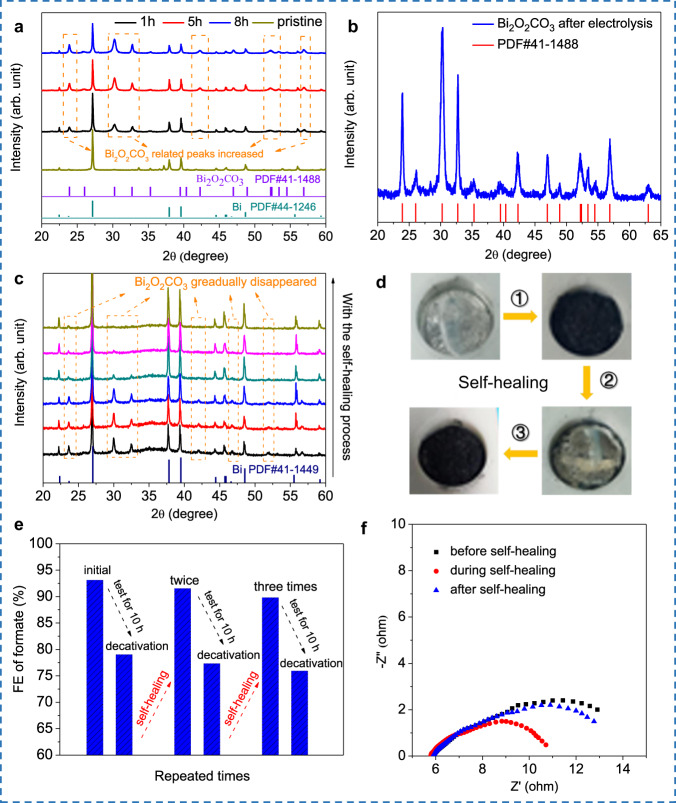
Structural and electrochemical data presentation related to self-healing process. **a** XRD patterns for bismuth catalyst loaded on LM surface during electrocatalytic CO_2_RR process. **b** XRD patterns for Bi_2_O_2_CO_3_ directly loaded on LM surface after 4 h of reaction. **c** In situ XRD patterns of Bi catalysts phase transforming during the self-healing. **d** Display of electrode surface state before and after self-healing in one cycle. **e** Three replicates of Faradaic efficiency healing after 10 h of reaction. **f** Electrochemical impedance spectrum (EIS) before and after self-healing. Source data are provided as a Source Data file.

The durability of the LM electrode was tested at −0.9 V as the formate product has the highest FE at this potential. During the whole electrolysis process, the *i*–*t* curve suffered a current density dropping from −15 to −5 mA cm^−^^2^ within 10 h, and the FE also decreased accordingly (Fig. 4d). To ascertain the cause of deactivation, catalysts on the LM electrode surface was collected after electrolysis. The XRD results detected a newly formed phase of Bi_2_O_2_CO_3_ in the bismuth catalyst. And as the electrolysis prolonged from 1 to 8 h, the contents of Bi_2_O_2_CO_3_ kept increasing (Fig. 5a). In reported literature, the generation of Bi_2_O_2_CO_3_ is a common phenomenon when active Bi continuously exposed to CO_2_ atmosphere in KHCO_3_ aqueous solution^29^, and it was ascribed to the reason for the deactivation of bismuth catalyst^48^. To ensure that Bi_2_O_2_CO_3_ can not be reduced on the LM surface, another LM electrode loaded with pure Bi_2_O_2_CO_3_ was tested. After 4 h of electrolysis, the Bi_2_O_2_CO_3_ still survived on the LM surface, and no bismuth was detected (Fig. 5b). To further confirm the by-product Bi_2_O_2_CO_3_ had no catalytic ability, the Bi_2_O_2_CO_3_ loaded LM electrode was used to LSV tests in KHCO_3_ saturated solution. As shown in Supplementary Fig. 15, the LSV curve of the Bi_2_O_2_CO_3_ loaded LM electrode showed a far worse catalytic ability than the Bi-loaded LM electrode.

Traditionally, for the catalyst on a rigid current collector (such as foamed metal or glassy carbon), phase transformation-induced deactivation is hard to overcome^3^. Here, in our LM system, such a problem can be easily solved by a self-healing process. To prove this, in situ XRD was adopted again to verify the phase healing of the catalytic system. The bismuth catalyst with the Bi_2_O_2_CO_3_ phase was dispersed into LM sea and regenerated by applying a voltage in the TPS mode (detailed in Method part: self-healing electrode). Although Bi_2_O_2_CO_3_ can not be electrochemically reduced on the LM surface, when it is dispersed into the LM again, the electric driving inside of the LM can promote the bismuth regeneration reaction. As shown in Fig. 5c, during the self-healing process, the intensity of main peaks for Bi_2_O_2_CO_3,_ which located at 23.9°, 30.3°, 32.7°, and 42.3° was, gradually disappeared. After the healing process, only Bi characteristic peaks are reserved. Figure 5d demonstrated the surface state of the LM electrode before and after the self-healing process in one cycle.

Figure 5e represented the FE during the healing, and Supplementary Fig. 16 showed the corresponding repeated current curves. During 10 h testing, although the FE and current density dropped gradually, they can recover to the original level after the self-healing process. Although it drops again in a subsequent process, repeatability makes it a reliable regeneration method. What is more, the impedance of the electrode can also recover after the healing process (Fig. 5f). Supplementary Fig. 17 illustrated the device employed in entire experiments, and Supplementary Fig. 18 demonstrated more cycles of the repeated *i*–*t* curves. In short, this Bi-based LM electrode demonstrated an ability of electrocatalytic CO_2_RR, with a feasible ability of self-healing.

## Discussion

We have developed a catalytic system with self-healing electrocatalysis ability, by successfully utilizing the solvent property of LM and an electrohydrodynamic-induced driven process. As a proof of concept, Bi_2_O_3_ as a catalytic precursor was dispersed in LM. When the negative potential was applied, the Bi_2_O_3_ was first reduced to Bi atoms dispersed in the LM as an intermediate state, and then converted to catalytic bismuth with controllable morphologies based on different electrohydrodynamic evolved pathways. This bismuth-based LM electrode showed a considerable catalytic ability of CO_2_RR in wide potential windows. When it suffered irreversible deterioration as conventional electrode did, the catalyst can be self-healed by simple re-dispersion followed by an electrochemical reconfiguration. Overall, this conception provides a unique but feasible proposal to design healable catalytic electrodes. By utilizing the conductivity, fluidity, flexibility, and maneuverable properties of LM, other catalytic elements can be integrated with LM, and endow it the application potential in various catalytic occasions in the future.

## Methods

### Materials

Gallium (Ga, ingot, purity: 99.99%) was purchased from Aladdin, China. Bi_2_O_3_ and KHCO_3_ were purchased from Xiya Reagent, and all the above reagents were used as received and without any purification. The self-supporting electrode cell to load LM is made of PLA texture and printed by a 3D printer, there is a tinned copper wire inside that connects to LM to keep the electrons transferred.

### Disperse of Bi_2_O_3_ in LM matrix

In a typical process, 0.2 g Bi_2_O_3_ and 4 g Ga were added in an agate mortar, simple physical mixing methods are used by grinding, heat slightly (~35 °C) to ensure that Ga remains liquid throughout the grinding process, and the grinding time is restrained within 1 min to control the oxidation degree of Ga. The whole system changed to show metallic luster again when Bi_2_O_3_ was uniformly dispersed into the LM matrix.

### CV diagnosis

The electrochemical diagnosis was used to preliminarily judge the electrochemical reactions happening in the LM electrode under the action of the electric field at ~30 °C. The Bi_2_O_3_–LM mixture, after grinding, was placed in a 3D printed cell directly as a working electrode, Ag/AgCl as a reference electrode, and Pt wire as a counter electrode. In an H-cell with 0.5 M KHCO_3_ as an electrolyte, the potential window was chosen from −1 V to 0 V, the scan rate was 100 mV s^−1^. CV was finished when the CV curves become stable (after 400 cycles); the potential used in CV diagnosis and other experiments in this study are all vs RHE and converted by the following formula: *E*(RHE) = *E*(Ag/AgCl) + 0.197 V + 0.0591× pH.

### Electrohydrodynamic-induced evolution to control the morphology of bismuth

Bi_2_O_3_–LM mixture was used as the cathode, platinum wire as the counter electrode, and Ag/AgCl as the reference electrode. In a three-electrode system composed of H-type cells, 0.5 M KHCO_3_ was employed as the electrolyte, and the two chambers are separated by nafion 117 membrane. Three modes of potential were applied by a Koster electrochemical workstation (310 M) during the evolution process: CVP (−1 V vs. RHE); POP (two configurations, POP-I exploited pulsing 10 s at −1 V and 50 s relaxation as a cycle; POP-II exploited pulsing at −1 V for 15 s then change to −0.6 V for 15 s as a cycle); TPS (scanning window was chosen from −1.6 V to −1.2 V vs. RHE with three different scan rate: 100, 60, and 20 mV s^−1^, respectively). All test conditions are controlled at ~30 °C.

### Electrocatalysis CO_2_RR process

Electrocatalysis CO_2_RR employed a similar apparatus as the CV diagnosis process used. Test conditions are controlled at ~30 °C. The electro-catalytic cell was strictly sealed. LSV was acquired on the evolved LM electrode in the cathode cavity with N_2_ or CO_2_-saturated atmosphere, with a potential window from −1.8 V to −0.8 V vs. RHE at a scan rate of 10 mV s^−1^. Faraday efficiency was obtained by precise analysis of the product by gas chromatography (Shimadzu GC 2014) and NMR (INOVA 400 MHz). All gas and liquid products are normalized. Electrochemical impedance was detected on CHI 760E at −1.2 V vs. RHE with a frequency from 0.01 to 100,000 Hz. *I*–*t* curves was tested at −0.9 V vs. RHE. All potentials given in the experiment were without iR compensation.

### Self-healing electrode

During the self-healing process, the electrode was taken out of the electrolyte, and then the physically stirred was introduced. The deactivated bismuth catalyst on the electrode surface will be dispersed into the Ga matrix again. After several minutes, the electrode surface re-appeared the metallic luster of Ga, confirming the deactivated bismuth was totally dispersed in LM. The electrode was placed still for another 30 min to mix well, then reduction potential (TPS from -0.6 to −1.2 V vs. RHE) was applied again. The self-healing process was finished after the fresh bismuth was segregated again. The above operation was repeated in the cyclic regeneration experiment with 0.2 g Bi_2_O_3_ and 4.5 g Ga, the electrolyte was updated every time during the testing process. Test conditions are controlled at ~30 °C.

### In situ Raman test

In situ Raman spectrum was performed at ~30 °C on an IS50/DXR2 (Thermo Fisher, USA) is equipped with a self-designed electrochemical module. The testing with a 532 nm laser source, focused on the surface of the LM electrode during CV scanning (the CV electrochemical window was chosen from −1 V to −0.6 V vs. RHE).

### In situ XRD for electrohydrodynamic evolution and self-healing tests

In situ, XRD was used to analyze the Bi species changing details during the electrohydrodynamic evolution. All the experiment was conducted on a D8 ADVANCE (Bruker, Germany). Equipped with a self-designed electrochemical module, curves were collected at a CV electrochemical window from −0.6 V to −1 V vs. RHE, the scan rate was kept at 50 mV s^−1^. In in situ XRD for self-healing test, the instruments used were the same as before, TPS mode was used again to drive the electrochemical reduction and healing of the bismuth catalyst. The tests were operated at ~30 °C.

## Supplementary information

Supplementary Information File

Description of Additional Supplementary Information file

Supplementary Movie 1.electrohydrodynamics induced evolution

Supplementary Movie 2 Constant voltage polarization-CVP

Supplementary Movie 3 Pulsing oscillation polarization-POP

Supplementary Movie 4 Triangle potential scanning-TPS

## Data Availability

All data that support the findings are available within the paper and its Supplementary information files. Source data are provided in this paper.

## Source data

Source Data

## Code availability

This study does not use any custom code or mathematical algorithm, and code availability is not a mandatory requirement for this work.
