# Supplementary material for: A self-healing electrocatalytic system via electrohydrodynamics induced evolution in liquid metal
Source: Nat Commun. 2022 Dec 9;13:7625. doi: 10.1038/s41467-022-35416-w (PMC9734151; doi:10.1038/s41467-022-35416-w)
Supplement: Supplementary file 1 — Supplementary Information File [file 41467_2022_35416_MOESM1_ESM.pdf]

Supplementary Information

A self-healing electrocatalytic system via electrohydrodynamics induced evolution in liquid metal

Yifeng Hou, Fengyan Wang, Chichu Qin, Shining Wu, Mengyang Cao, Pengkun Yang, Lu Huang, Yingpeng Wu*

State Key Laboratory of Chem/Bio-Sensing and Chemometrics, Advanced Catalytic Engineering Research Center of the Ministry of Education, College of Chemistry and Chemical Engineering, Hunan University, Changsha, 410082, P. R. China.

*Corresponding Author

E-mail addresses: wuyingpeng@hnu.edu.cn (Y. P. Wu)

Supplemental Figures and Supplemental Tables.

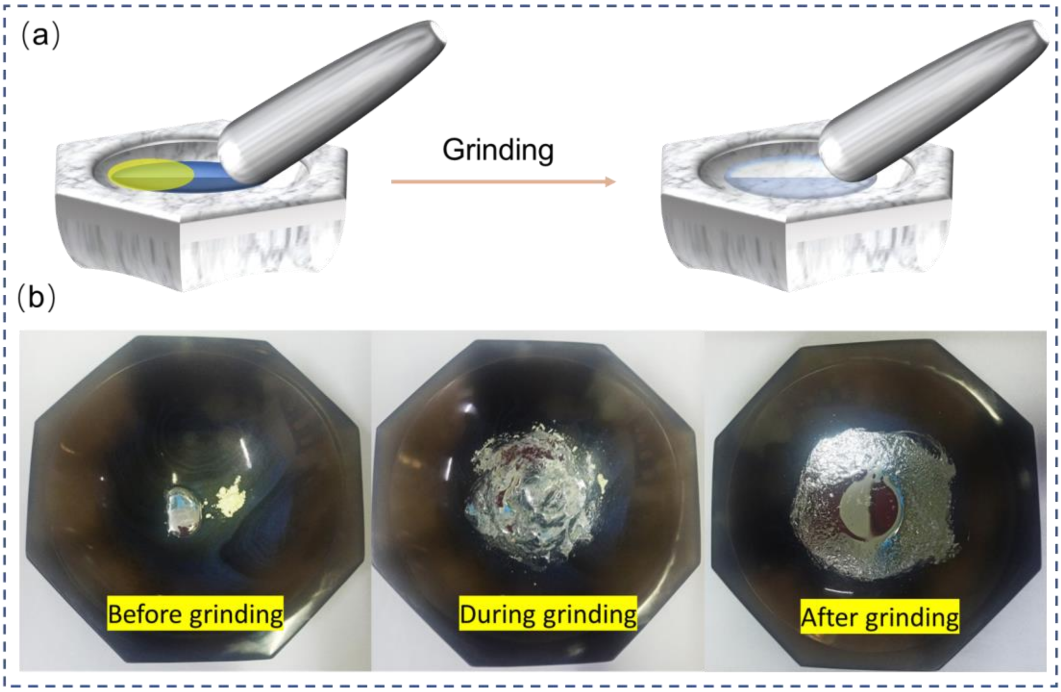

Supplementary Fig. 1 The preparation process of Bi based liquid metal electrode. (a) Schematic illustration of the grinding process for producing Bi_2O_3-LM mixture. (b) Photos of the Bi_2O_3-LM mixture before grinding, during grinding and after grinding respectively. When grinding process finished, the mixture became fluid and reflects metallic luster.

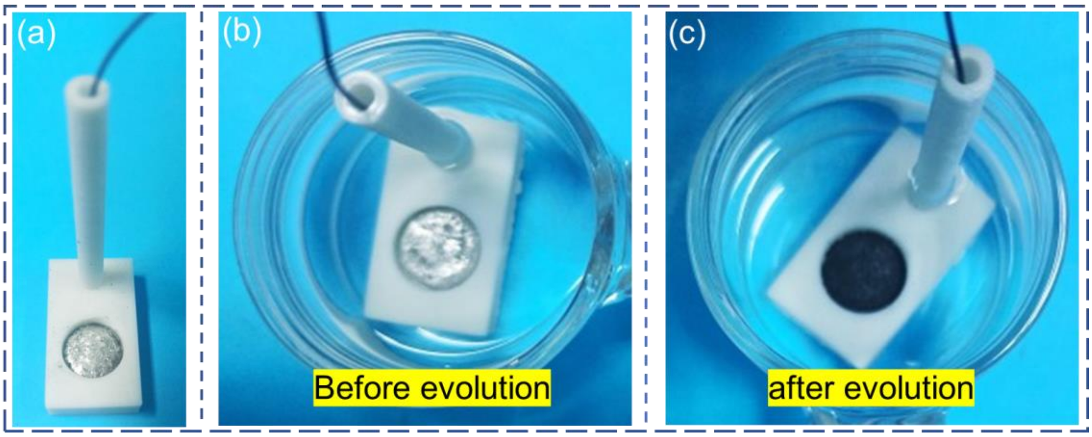

Supplementary Fig. 2 Physical display of Bi based LM electrode. (a) The 3D printed electrolytic cell with loaded Bi_2O_3-LM mixture. (b) The surface state display of LM electrode before and (c) after evolution.

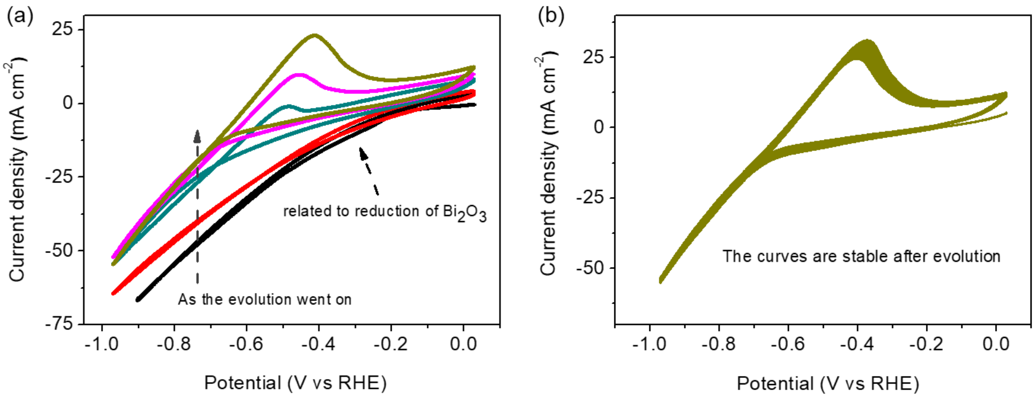

Supplementary Fig. 3 The CV diagnosis curves in 0.5 M $KHCO_3$ solution on Bi_2O_3-LM mixture within a potential window from -1 V to 0 V vs RHE. (a) Gradually increased CV curves (b) Stabilized CV curves after cycling.

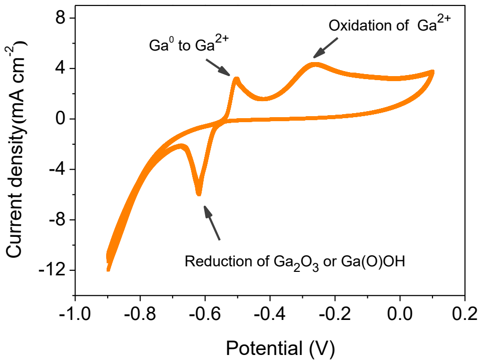

Supplementary Fig. 4 The CV curves of pure Ga in 0.5 M $KHCO_3$ aqueous solution. the peaks represent oxidation of Ga^0 to Ga^{2+}, oxidation of Ga^{2+} and reduction of Ga_2O_3 or Ga(O)OH, respectively.

According to the published literature, the two oxidation peaks are attributed to the stepwise oxidation of Ga (Ga^0 to Ga^{2+} and oxidation of Ga^{2+}). And the reduction single peak is attributed to the reduction of Ga oxides[1, 2].

The analysis for possible electrochemical reactions according to Supplementary Fig. 3 and 4:

As Bi_2O_3 was dispersed in LM matrix, the changes of CV curves should be related to both Ga and Bi_2O_3. As shown in Supplementary Fig. 3a, the CV curves were gradually shifted upwards, this decreased electrochemical signal demonstrated the gradually electrochemical reduction of Bi_2O_3. The decrease of Bi_2O_3 in LM matrix should be responsible for the decay of the current. When all of the Bi_2O_3 was consumed, the reduction process completed, the CV curves became stable (Supplementary Fig. 3b). After cycling, the oxide peaks became stable at -0.4 V (Supplementary Fig. 3b), it should be attributed to the oxidation of Ga, as pure LM Ga exhibited same trend (Supplementary Fig. 4).

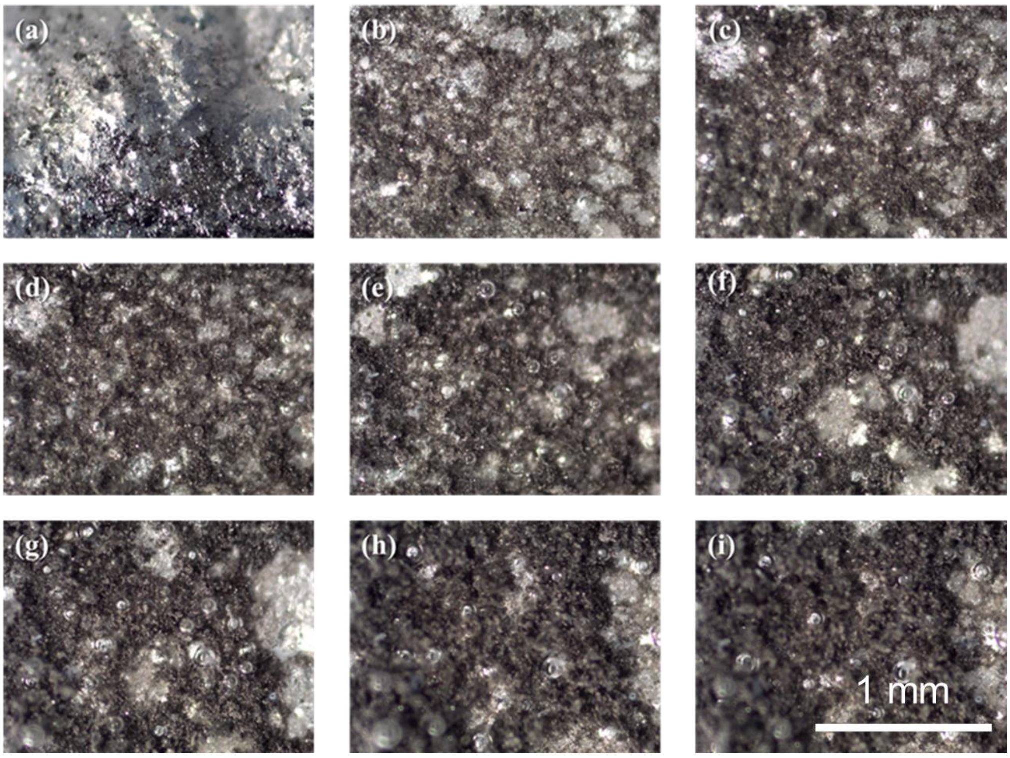

Supplementary Fig. 5 The surface segregation of LM during electro-hydrodynamic induced evolution captured by microscopy according Movie 1. From (a) to (i), the loaded black species on the LM surface gradually increased due to the continuous segregation.

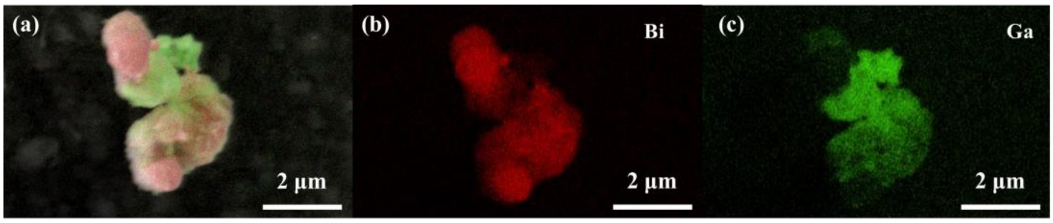

(d)

Element	Atomic %	Atomic % Error	Weight %	Weight % Error
Ga	55.2	0.4	29.2	0.2
Bi	44.8	0.3	70.8	0.5

Supplementary Fig. 6 The SEM elemental mappings of Bi coated Ga particles after CV diagnose. (a)-(c) Bi-Ga (green and red), Bi (red) and Ga (green). (d) The element ratio of Ga and Bi.

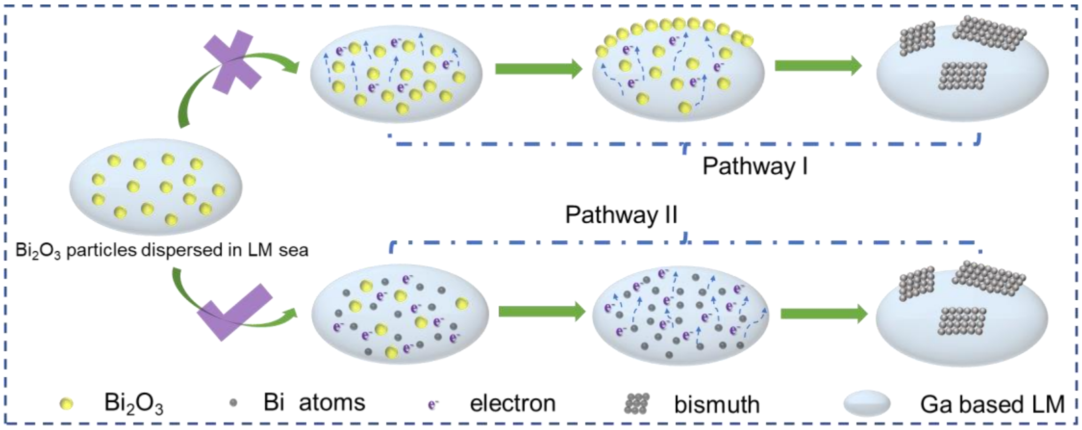

Supplementary Fig. 7 Two assumed evolution pathways of Bi species under electric field in LM. In pathway I, Bi_2O_3 was migrated from the matrix to LM surface by electric driving, then reduced to crystalline bismuth. In pathway II, Bi_2O_3 transformed into highly dispersed Bi atoms, then the dispersed Bi atoms migrated from the inside of LM driven by electric field and segregated on LM surface, forming crystal bismuth.

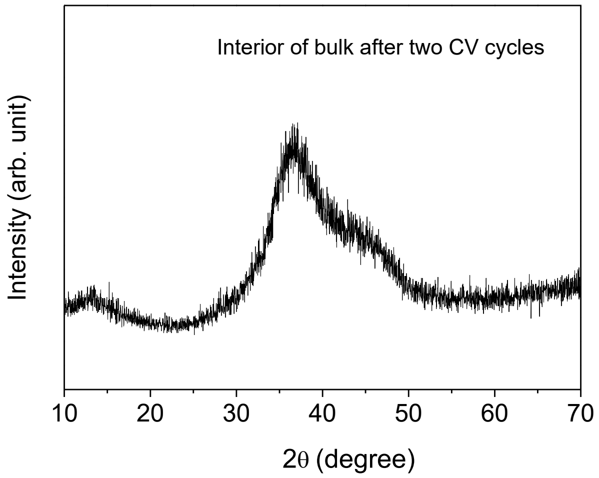

Supplementary Fig. 8 The XRD characterization of the dissected LM electrode after only two CV cycles. No Bi_2O_3 or Bi characteristic peaks were detected, which proved that Bi_2O_3 has already reduced to highly dispersed Bi atoms.

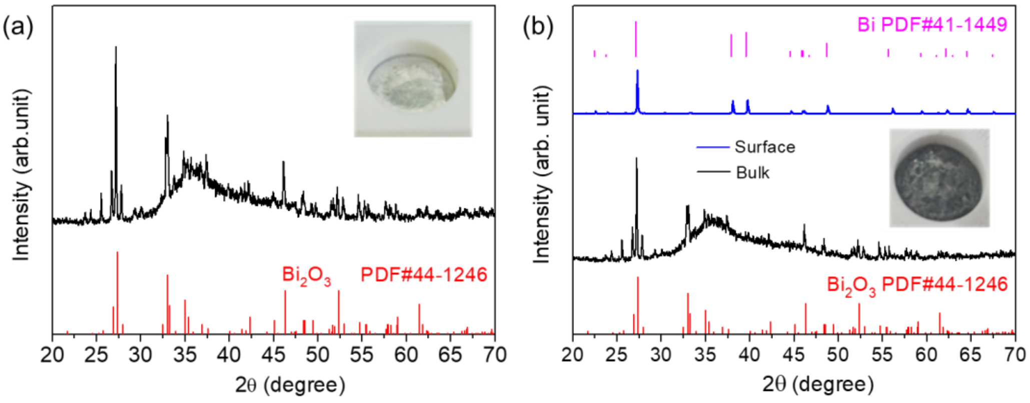

Supplementary Fig. 9 The XRD characterization of dissected Bi_2O_3-LM mixture placed in air and in $KHCO_3$ solution. (a) No change occurred on surface or in matrix of the LM, confirming no spontaneous replacement happened in this situation. (b) A thin layer of Bi appears on the surface of the LM which was placed in $KHCO_3$ solution. The Bi_2O_3 in the LM matrix remains unreduced, and Bi appears only on the surface.

The analysis for Supplementary Fig. 9 and the possible galvanic replacement in LM matrix:

Considering that the reduction potential of Bi is more positive than that of Ga, the galvanic replacement may occur in LM at the same time. Two supplemented experiments were conducted: (1) Ga electrode mixed with Bi_2O_3 was placed still in air. (2) Same preparation method but placed in $KHCO_3$ solution. In these two conditions, without additional negative potential, the replacement only contributed by electronegativity of pure Ga. After staying for 10 hours, the control group placed in air exhibited no changes on the surface (insert graph, Supplementary Fig. 9a). The XRD characterization of dissected LM confirmed that the Bi_2O_3 existed in LM matrix was not spontaneously reduced to Bi (Supplementary Fig. 9a). This proved the spontaneous replacement inside of LM matrix is hard to happen.

For the control group in $KHCO_3$ solution, the phenomenon was different but explainable: we noticed that a thin black layer appeared on LM surface, and

the reaction rate was far less than the electrochemical driving process. The products were confirmed as bismuth by XRD (Supplementary Fig. 9b). The formation rate of this bismuth is ~1.25 mg/h, while in the case of electrochemical driving, this value is 85 mg/h, 67 times higher than the spontaneous process. What's more, without the electrochemical driving force, the XRD of dissected LM droplet showed that of Bi_2O_3 still existed in the LM matrix, and no bismuth can be detected in LM. (Supplementary Fig. 9b).

We can draw the following conclusions based on the above facts: 1. Galvanic replacement does exist, but only occurred at the interface of the LM-$KHCO_3$ solution, 2. The rate of galvanic replacement is very slow, which can be ignored comparing with the electrohydrodynamic induced evolution and 3. This interface process is completely different from the electrohydrodynamic induced evolution in LM presented in our paper.

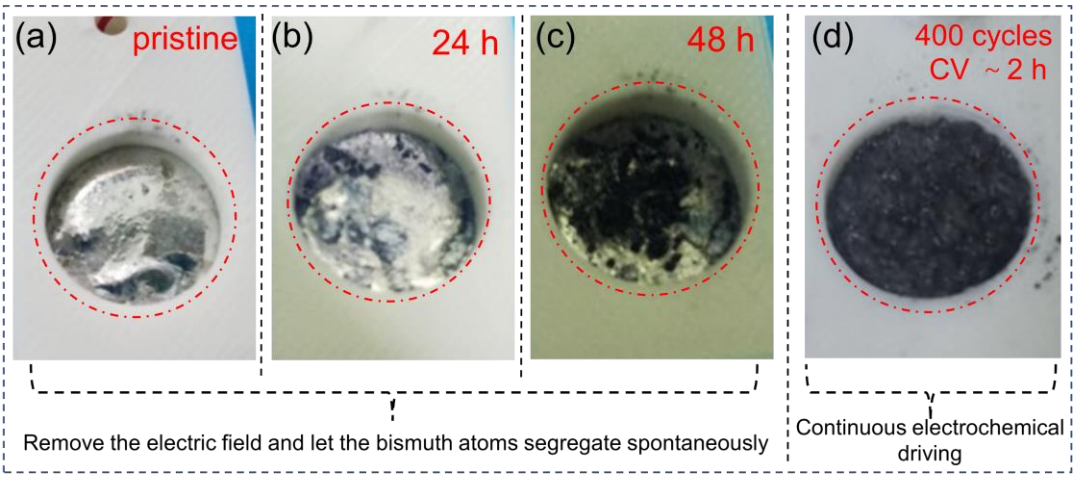

Supplementary Fig. 10 The compared images of the electrode surface state resulted by only two cycles or continuous driving. (a) The pristine state after two CV cycles then removed the electric field. (b) The state for (a) placed 24 h. (c) The state for (a) placed 48 h. (d) The state after electrohydrodynamic continuous driving.

There are only slight black spots appeared on LM surface in 48 h in former situation (remove the electric field and let the bismuth atoms segregate spontaneously). The bismuth yield was far less than the results caused by electrohydrodynamic continuous driving.

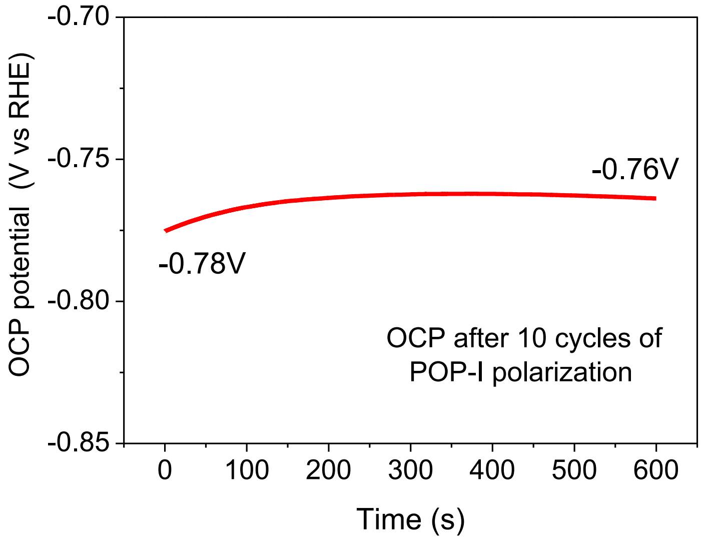

Supplementary Fig. 11 The OCP of LM electrode after 10 cycles of POP-I. the potential only changed a little (from -0.78 V to -0.76 V) within 600 seconds.

The discussion of OCP of LM electrode:

Supplementary Fig. 11 demonstrated the OCP of LM electrode after 10 cycles of POP-1 then removed the external voltage. The OCP of LM only showed a little decrease from -0.78 V to -0.76 V within 600 s. This means that although the electric field was removed, the electrode potential of the LM changed a little, which further resulted in no significant changing of the LM surface energy.

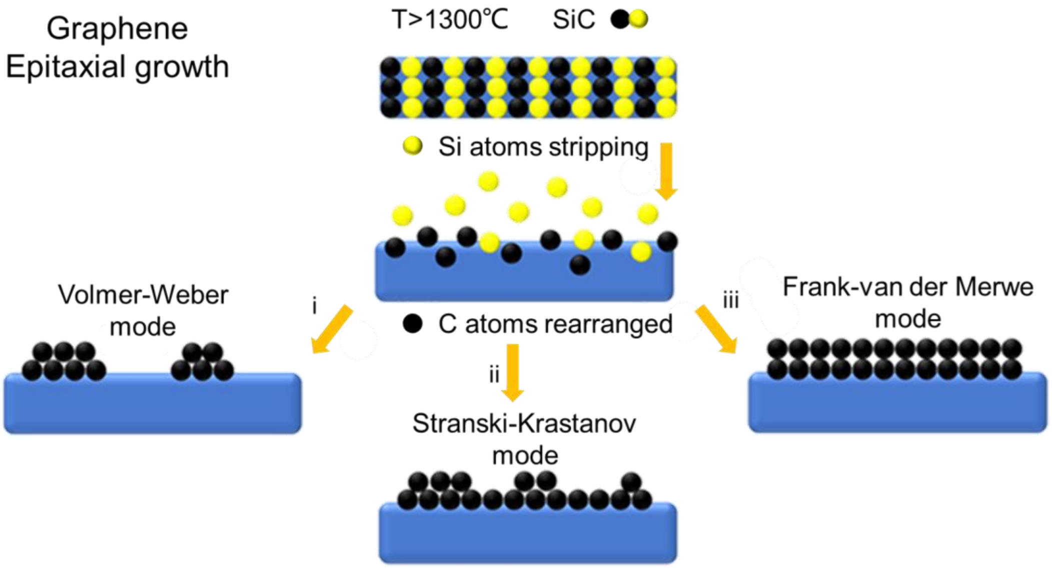

Supplementary Fig. 12 The schematic illustration for graphene epitaxial growth. i Volmer-weber mode. ii Stranski-Krastanov mode. iii Frank-van der Merwe mode[3].

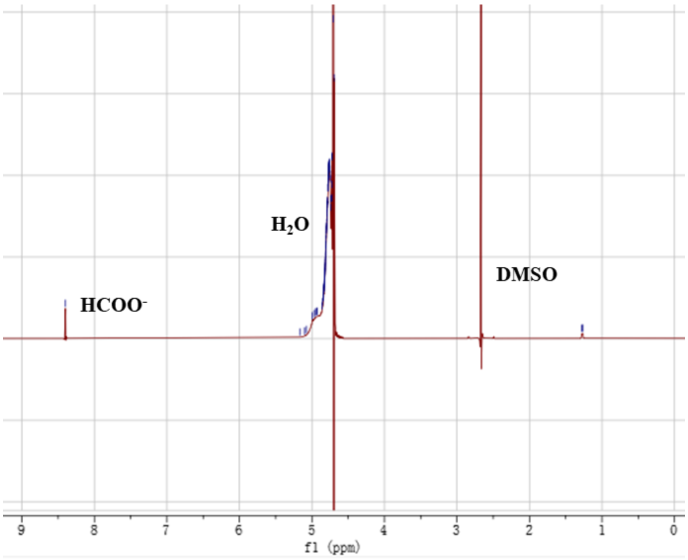

Supplementary Fig. 13 Representative NMR spectra for the electrochemical CO_2 reduction products analyzing. The peaks represent $HCOO^-$, H_2O and DMSO, respectively.

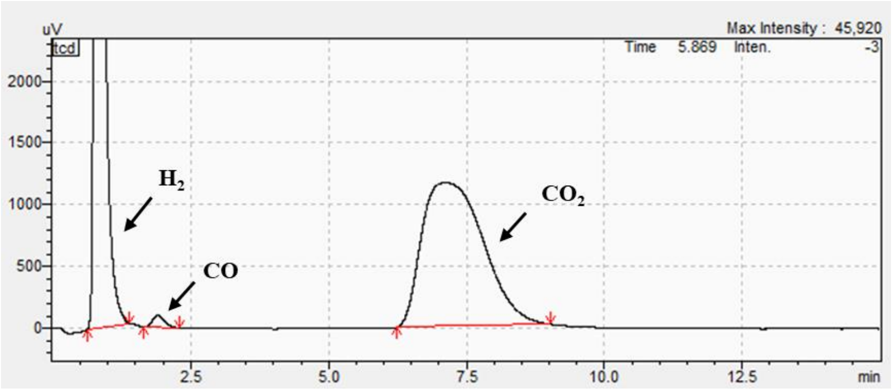

Supplementary Fig. 14 Representative GC results in TCD detector. The peaks represent H_2, CO and CO_2, respectively.

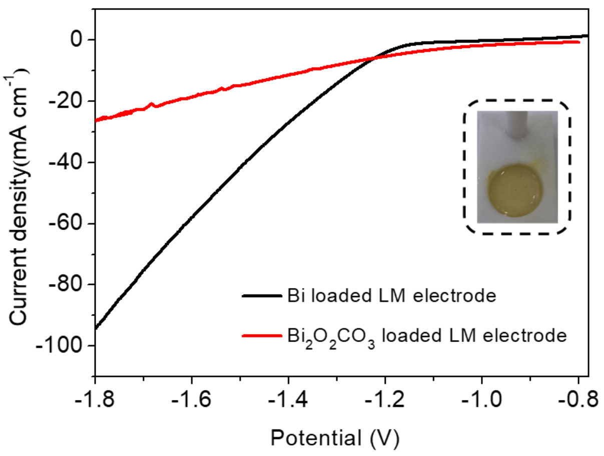

Supplementary Fig. 15 The contrast LSV curves for $Bi_2O_2CO_3$ and Bi loaded LM electrode in CO_2 saturated 0.5 M $KHCO_3$ electrolyte. Insert image: Schematic diagram of $Bi_2O_2CO_3$ loaded LM electrode after electrolysis.

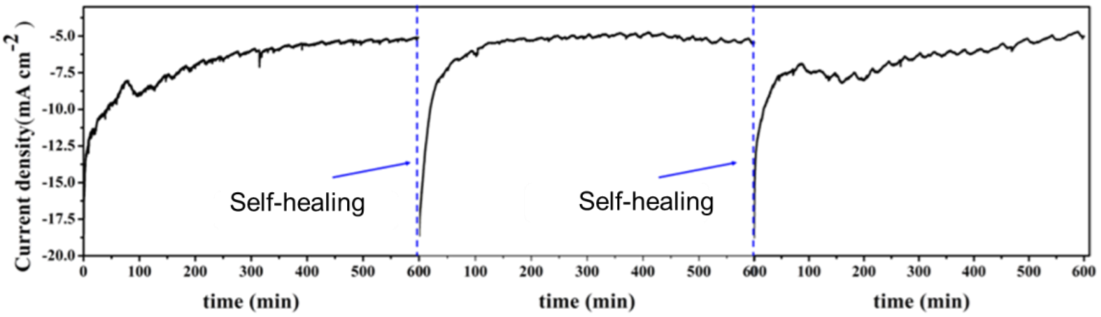

Supplementary Fig. 16 The corresponding repeated current curves for Faradaic efficiency. The current density decayed as the catalyst suffered long time of service. However, after self-healing, the current recovered significantly. Although the subsequent decay was difficult to avoid, the cyclability of healing makes it a reliable method.

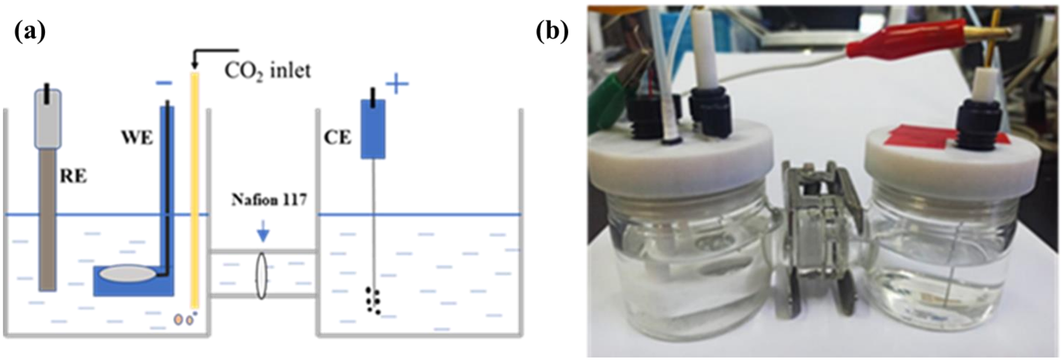

Supplementary Fig. 17 Illustration of the testing H cell (a) Schematic and (b) physical devices for electrochemical diagnostics and electrochemical CO_2RR.

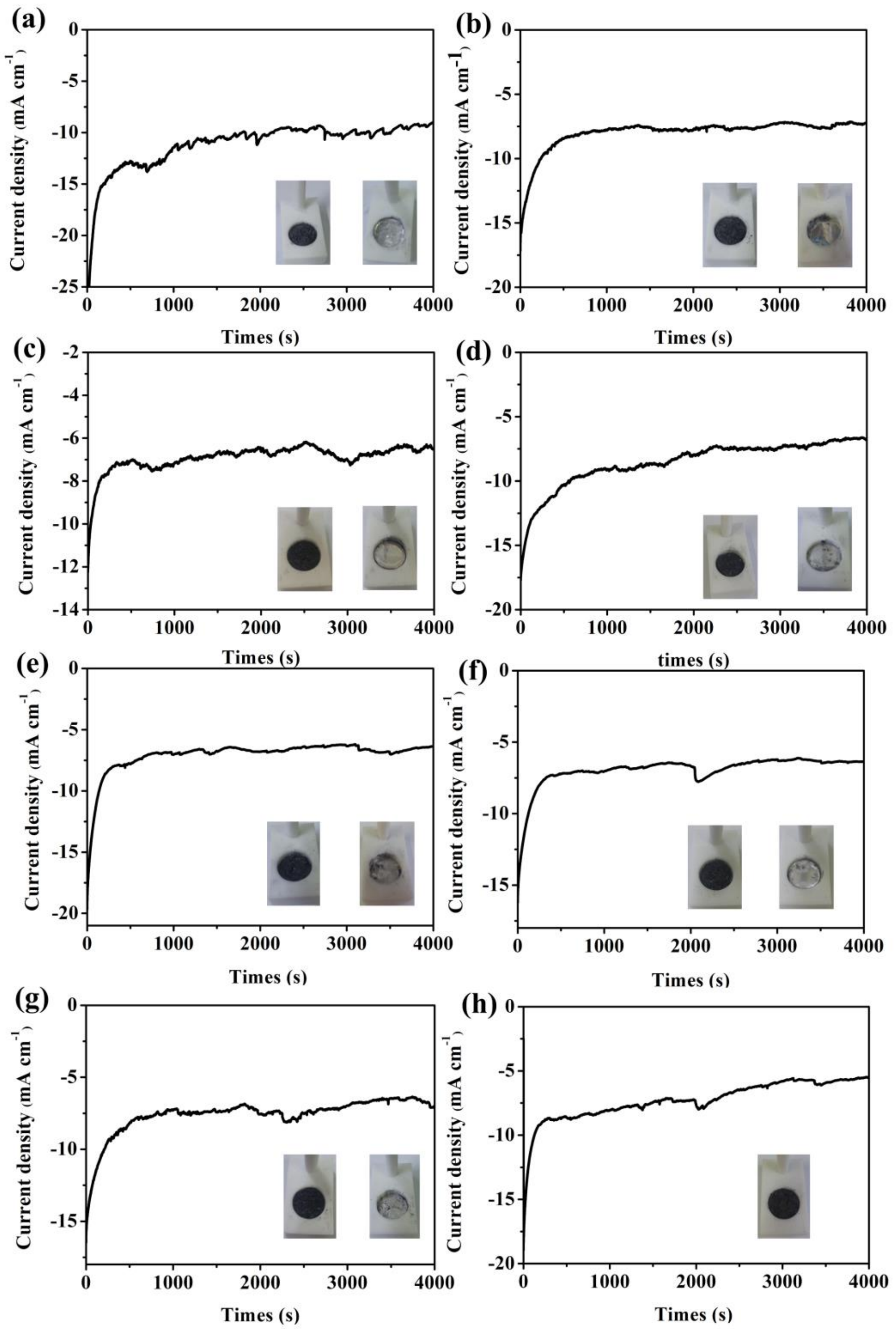

Supplementary Fig. 18 The display of self-healing ability of LM system for eight replicates. The inset images show bismuth loaded on LM surface (left) and re-dispersed (right) into the LM after mechanical stirring.

Reference

1. Song M., Daniels K. E., Kiani A., Rashid-Nadimi S. & Dickey M. D. Interfacial Tension Modulation of Liquid Metal via Electrochemical Oxidation. *Adv. Intell. Syst.* **3**, (2021).
2. Mayyas M. et al. Pulsing liquid alloys for nanomaterials synthesis. *ACS Nano* **14**, 14070-14079 (2020).
3. Kaiser N. Review of the fundamentals of thin-film growth. *Appl. Optics* **41**, 3053-3060 (2002).
