# Supplementary material for: A self-healing electrocatalytic system via electrohydrodynamics induced evolution in liquid metal
Source: Nat Commun. 2022 Dec 9;13:7625. doi: 10.1038/s41467-022-35416-w (PMC9734151; doi:10.1038/s41467-022-35416-w)
Supplement: Supplementary file 2 — Description of Additional Supplementary Information file [file 41467_2022_35416_MOESM2_ESM.pdf]

Description of Additional Supplementary Files (Supplementary Movies)

File name: Supplementary Movie 1

Description: Video of electrohydrodynamics induced Bi species evolution in LM matrix and loaded on LM surface eventually.

File name: Supplementary Movie 2

Description: Specific evolution mode-Constant Voltage Polarization (CVP). This mode will not cause surface energy changes.

File name: Supplementary Movie 3

Description: Specific evolution mode-Pulsing Oscillation Polarization (POP). This mode will cause surface energy changes suddenly.

File name: Supplementary Movie 4

Description: Specific evolution mode-Triangle Potential Scanning (TPS). This mode will cause surface energy changes in a continuous way.
